# Tuning the Shades of Red Emission in InP/ZnSe/ZnS Nanocrystals with Narrow Full Width for Fabrication of Light-Emitting Diodes

**DOI:** 10.1021/acsomega.3c05580

**Published:** 2023-10-13

**Authors:** Ehsan Soheyli, Ayşenur Biçer, Sultan Suleyman Ozel, Kevser Sahin Tiras, Evren Mutlugun

**Affiliations:** † Department of Electrical-Electronics Engineering, Abdullah Gül University, Kayseri 38080, Türkiye; ‡ Department of Physics, Faculty of Sciences, Erciyes University, Kayseri 38030, Türkiye

## Abstract

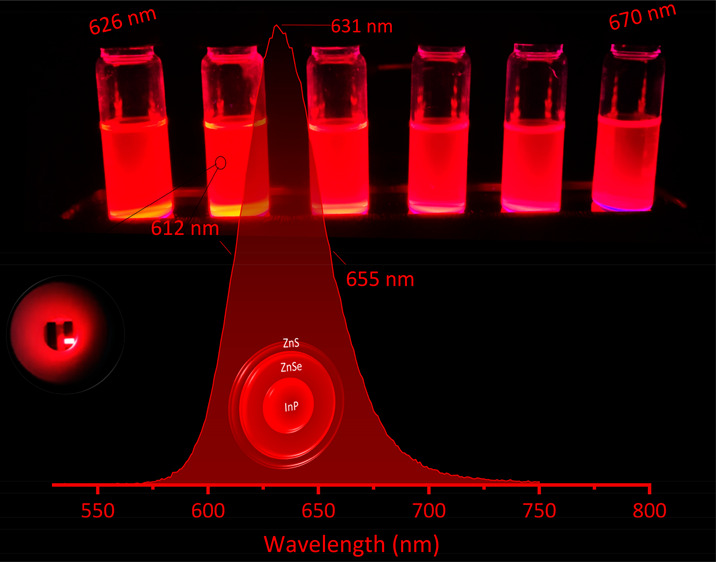

While Cd-based luminescent nanocrystals (NCs) are the most mature NCs for fabricating efficient red light-emitting diodes (LEDs), their toxicity related limitation is inevitable, making it necessary to find a promising alternative. From this point of view, multishell-coated, red-emissive InP-based NCs are excellent luminescent nanomaterials for use as an emissive layer in electroluminescent (EL) devices. However, due to the presence of oxidation states, they suffer from a wide emission spectrum, which limits their performance. This study uses tris(dimethylamino)phosphine (3DMA-P) as a low-cost aminophosphine precursor and a double HF treatment to suggest an upscaled, cost-effective, and one-pot hot-injection synthesis of purely red-emissive InP-based NCs. The InP core structures were coated with thick layers of ZnSe and ZnS shells to prevent charge delocalization and to create a narrow size distribution. The purified NCs showed an intense emission signal as narrow as 43 nm across the entire red wavelength range (626–670 nm) with an emission quantum efficiency of 74% at 632 nm. The purified samples also showed an emission quantum efficiency of 60% for far-red wavelengths of 670 nm with a narrow full width of 50 nm. The samples showed a relatively long average emission lifetime of 50–70 ns with a biexponential decay profile. To demonstrate the practical ability of the prepared NCs in optoelectronics, we fabricated a red-emissive InP-based LEDs. The best-performing device showed an external quantum efficiency (EQE) of 1.16%, a luminance of 1039 cd m^–2^, and a current efficiency of 0.88 cd A^–1^.

## Introduction

The recent signs of progress in the solution-processed fabrication of light-emitting diodes (LEDs) and light-emitting panels have been the preparation of highly luminescent colloidal nanocrystals (NCs) with narrow photoluminescence (PL) profiles at green and red emission wavelengths. The industry is currently looking for cost-effective, less toxic, and physicochemically efficient NCs in order to engage them in commercialization. From this point of view, InP-based NCs are one of the most demanding types of luminescent NCs because they show better biofriendly properties than other NCs, such as Cd- and Pb-based perovskite NCs. InP-based NCs also show optoelectronic results that are far better their carbon-based counterparts, which typically have external quantum efficiencies (EQEs) that are less than 5%.^1^ However, despite the great achievements of green- and blue-emissive InP-based NCs in high-performance LEDs,^2,3^ reaching the longer wavelength emissions in the red region (typically >620 nm) with an emission profile narrower than 50 nm is challenging due to the difficulties accompanying the growth of these NCs. The main reason is the covalent nature of the chemical bonds in InP NCs.^4^ This is even more challenging if one is looking for a narrow full width at half-maximum (fwhm) emission profile in the deep-red region of the spectrum.

Several physicochemical deficiencies limit or at least decelerate the progress of red-emissive InP-based NCs for commercialization: (I) the core-only InP NCs show weak and quite unstable luminescent characteristics; (II) the nucleation rate of InP cores is very fast, and the growth process to the desired size (typically >3.5 nm) leads to an increase in the size distribution, which has a negative effect on the fwhm of the PL profile; and (III) the colloidal synthesis process has to be performed via complicated, time-consuming, two-pot processes, which are not appropriate for the upscaling and reproducibility requirements of commercialization. In this regard, various attempts have been carried out with either one-pot methods like the use of zinc-oxo clusters,^5^ a thick intermediate ZnSe (or ZnMnS) shell layer,^6,7^ hydrofluoric acid (HF),^8^ and ligand-assisted post-treatment^9^ or two-pot methods like ZnF_2_-assisted synthesis,^10^ aminophosphine-derived halide-assisted synthesis,^11^ and stoichiometry control.^12^ Several well-established studies have also focused on combining these different chemical tricks to reach stable, intense, pure-red emissions for better device performance.^13−15^ Among these strategies, using the benefits of surface fluorination to remove the oxidation states on the surfaces of InP core NCs is a promising method, usually done with HF.^16−18^ The common colloidal synthesis approach employed in all of these studies is hot-injection, which acts as a powerful tool that provides nice control over the physicochemical properties of three-dimensionally confined quantum dots (QDs). The experimental parameters in this method can be easily tuned to induce a change to a particular target, such as a blue- or red-shift in the optical features, along with the possibility of large-scale synthesis. However, regardless of the synthesis method used, the inevitable part of each InP-based QD study is coating the core with a multishell layer, which includes a ZnSe intermediate layer to reduce lattice strain^19^ and surface/interface defects^20^ (the lattice mismatches of InP–ZnS, InP–ZnSe, and ZnSe–ZnS are 7.7%, 3.3%, and 4.4%, respectively). The colloidal hot-injection strategy performed in an oxygen-free medium is the best for this purpose. There is another problem regarding the limitations of the phosphor precursors. Tris(trimethylsilyl)phosphine (3TMS-P), the most conventional P precursor for preparing InP QDs, is highly toxic, expensive, and reactive, and it is consumed quickly upon injection into the In-containing solution. Hence, it is difficult to prepare larger InP QDs, as it is necessary to slowly add an extra stock solution of InP in order to govern the further growth of InP core QDs. This also requires a higher amount of 3TMS-P, which is not cost-effective and also limits reproducibility. From this point of view, tris(dimethylamino)phosphine (3DMA-P) is an appropriately cheaper P source with easier accessibility to longer wavelengths.^21^

Currently, the interest in one-pot colloidal methods is greater due to their simplicity, better reproducibility, and faster reactions. However, for almost all of the QDs created with these methods (even the highly luminescent QDs), their PL signals are located at wavelengths ≤620 nm or their fwhm is ≥50 nm,^6,8,15,17,22−24^ which both reduce the required red color purity for high-performance devices. As mentioned previously, this challenge is more dominant for far-red emissions at longer wavelengths, typically >640 nm. Recently, Huang et al. prepared multishell InP/ZnSe/ZnSeS/ZnS QDs with a bright deep-red emission at 670 nm.^25^ However, the PL profile was quite broad, showing an fwhm of 66 nm, which further demonstrates the importance of reaching narrow emissions at the deep-red region for InP-based NCs.

Having access to the entire red region of the visible spectrum with a narrow emission and high emission efficiency makes it possible to use suitable InP-based QDs in an LED structure with a desired band offset. This work demonstrates such InP-based QDs with tunable shades of red emission. In the present study, a colloidal one-pot/hot-injection method is developed to prepare InP/ZnSe/ZnS QDs. In this work, (I) a straightforward one-pot method with cheap precursors was used to realize InP-based QDs on a large scale (suitable for industry). (II) The experimental parameters were intentionally changed to make the emission tunable across the red region of the visible spectrum. (III) Large amounts of ZnCl_2_ and oleylamine (OLA) were used to form an efficient ZnSe shell around the core QDs and to reduce the nucleation rate (to provide better control over the fast nucleation of InP core QDs), respectively. OLA has multifunctional properties as a solvent, surfactant, and reducing agent, giving it the ability to minimize the formation of oxidized states, thus assisting with the uniform growth of the core. (IV) HF processing was performed in the first two shell layers to eliminate the surface oxide impurities between the interfaces. (V) A thick intermediate ZnSe shell was used to prohibit the leakage of the electron wave function and the interactions of the excitonic states with the surface of the NCs. These approaches resulted in a tunable emission at red-colored wavelengths (from 626 to 670 nm) with a pristine fwhm as narrow as 44 and 43 nm after a size selection process. The highest recorded photoluminescence quantum efficiency (PLQE) was 74% for 632 nm and, even more interesting, 60% for deep-red at 670 nm with a narrow emission of 50 nm, which are excellent results. Compared to the well-known Cd-based NCs and perovskite NCs, it is always challenging to investigate more environmentally friendly InP-based QD-LEDs (QLEDs) due to the difficulties in synthesizing high-quality materials. In this study, the synthesized InP/ZnSe/ZnS QDs were used to fabricate QLEDs. Our best device exhibited an EQE of 1.16%, a luminance of 1039 cd m^–2^, and CIE (*x*, *y*) coordinates of (0.69, 0.30) located at the deep-red region.

## Experimental Section

### Materials

InCl_3_ (98%), ZnCl_2_ (≥98% anhydrous, reagent grade), zinc stearate (ZnSt, purum, 10–12% Zn basis), 1-octadecene (ODE, technical grade, 90%), OLA (technical grade, 70%), oleic acid (OA, technical grade, 90%), tris(dimethylamino)phosphine (3DMA-P, 97%), hydrofluoric acid (HF, 48%), Se (99.99%), S (99.98%), trioctylphosphine (TOP, 97%), tetramethylammonium hydroxide pentahydrate (TMAH, ≥ 97%), zinc acetate dihydrate (99.999%), and 1-octanethiol (OT, ≥ 98%) were purchased from Sigma-Aldrich and used as received. Dimethyl sulfoxide (DMSO) was purchased from Merck. Ethanol, *n*-hexane (≥98%), and acetone (99.5%) were provided by Tekkim. For the fabrication of LEDs, ITO (indium tin oxide, Kaivo), PEDOT:PSS (poly(3,4-ethylenedioxythiophene):poly(styrene sulfonate), AI4083), PVK (poly(9-vinyl carbazole, Sigma-Aldrich), and Al (aluminum, Angstrom Engineering) were used as received.

### Stock Solutions

- 1Se-TOP was prepared by dissolving 3.7 mmol of Se powder in 3.1 mL of TOP.
- 2Se-TOP was prepared by dissolving 4.2 mmol of Se powder in 3.1 mL of TOP.
- 3Se-TOP was prepared by dissolving 5.4 mmol of Se powder in 3.1 mL of TOP.
- 4Se-TOP was prepared by dissolving 6.7 mmol of Se powder in 3.1 mL of TOP.
- S-TOP was prepared by dissolving 3 mmol of S powder in 3.1 mL of TOP.
- HF-acetone was prepared by mixing 65 μL of HF in 435 μL of acetone (HF should be handled carefully).
- ZnCl_2_-OLA was prepared by dissolving 1 mmol of ZnCl_2_ in 2 mL of OLA.
- ZnSt-ODE was prepared by dissolving 7.1 mmol of ZnSt in 17 mL of ODE.
- ZnOAc precursor was prepared by dissolving 1.1 mmol of ZnOAc and 1.6 mmol of OA in 2 mL of ODE.

### Synthesis Method

**Scheme 1:**
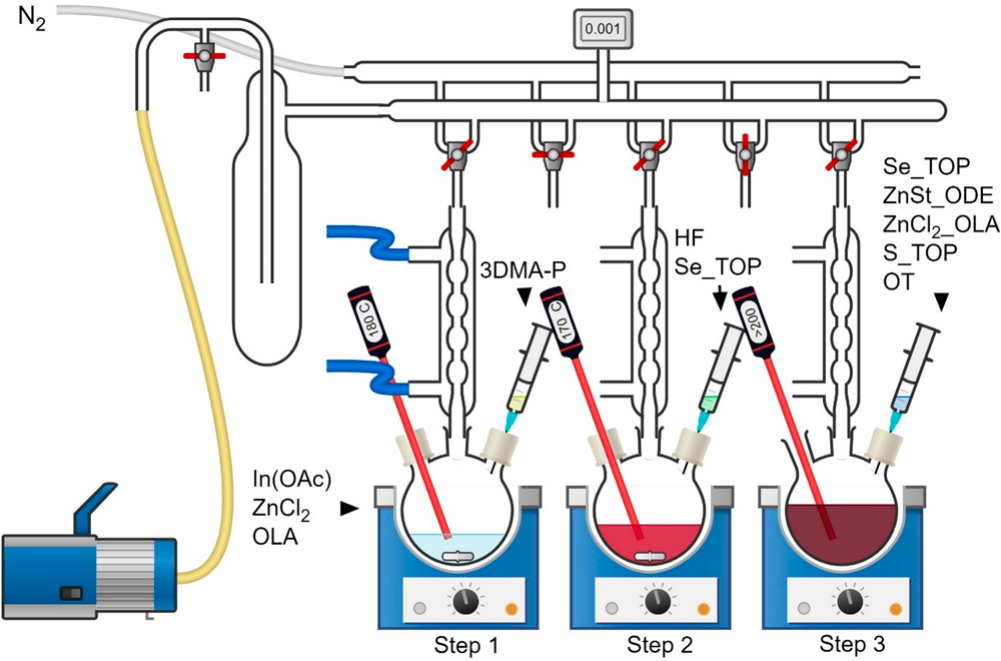
Synthesis Method for the Preparation of the InP/ZnSe/ZnS QDs

The details of the synthesis processes are summarized in Table 1, and a schematic explanation is given in Scheme 1. The method contains three steps: core synthesis, HF injection, and shelling process. Typically, InCl_3_, ZnCl_2_, and OLA were mixed inside the glovebox (the amount of InCl_3_ was 0.67 mmol). Then, the mixture was transferred to a three-neck flask equipped with a rubber septa and a thermocouple and connected to the condenser. The mixture was degassed at 120 °C for 30 min while being vigorously stirred in order to completely dissolve the powders. Backfilled with N_2_, the mixture was heated to 180 °C, where 2.40 mmol of 3DMA-P was quickly injected into the stirred solution, where it remained for the desired reflux time (Step 1). Immediately after injection, the color of the solution changed to red and then to dark-red. Next, the reaction cooled to 150 °C, and after a short time, it was heated again to 285 °C. When the mixture reached 170 °C, the HF precursor and 1 mL of 1Se-TOP were injected (Step 2). Then, at 220 °C, 1.5 mL of ZnCl_2_-OLA and 4 mL of ZnSt-ODE were co-injected. The reaction continued at 285 °C for 15 min. Then, 50 μL of the HF precursor, 1 mL of 2Se-Top, and 5 mL ZnSt-ODE were added, and the mixture was stirred for 30 min. For the third and fourth ZnSe shells, 1 mL of 3Se-Top and 1 mL of 4Se-Top were injected at 295 °C (and then stirred for 30 min) and 305 °C (and then stirred for 60 min), respectively. In these steps, 6 mL of ZnSt-ODE was also injected each time. To overcoat the QDs properly, the outer shell layers of ZnS were provided as follows. First, the reaction was heated to 310 °C, and 1 mL of the S-TOP precursor was quickly injected, followed by the addition of the ZnOAc precursor. The reaction continued for an extra 45 min. Finally, the reaction was cooled to 210 °C, 0.6 mL of OT was added dropwise, and the reaction continued for 1 h (Step 3). In the end, the reaction rapidly cooled to room temperature. To remove the unreacted species, hexane was added and the mixture was centrifuged at 4000 rpm. The precipitated parts were discarded. Then, a suitable mixture of ethanol/acetone was added to the clear solution of the QDs to precipitate the QDs; this was followed by centrifugation at 10 000 rpm for 10 min. This process was repeated one more time, and the final purified samples were dried in the lab atmosphere. It should be noted that, in order to make a reasonable change in shading the red emission (i.e., change in optical properties), we had to simultaneously modify several factors like what we did for RQ3 and RQ4: we simultaneously decreased the reaction time (core synthesis) from 17 to 12 min and increased the amount of ZnCl_2_ (from 3.3 to 3.87 mmol) (see Table 1).

**Table 1:** Details of the experimental parameters used to change the optical properties of the purified InP/ZnSe/ZnS QDs

code	InCl_3_ (mmol)	ZnCl_2_ (mmol)	OLA (mmol)	*t*_R_ (min)	HF:acetone (μL)/temp injection (°C)	UV/PL position (nm)	average size (nm)	PLQE (%)	fwhm (nm)
RQ1	0.67	3.3	3.04	20	0	642/670	10.5 ± 0.2	60	50
RQ2	0.67	3.3	3.04	20	60/270	631/666	10.3 ± 0.2	66	52
RQ3	0.67	3.3	3.34	17	^1^170/170	621/654	10.6 ± 0.1	68	47
					^2^50/285				
RQ4	0.67	3.87	3.34	12	^1^170/170	611/642	10.7 ± 0.1	71	45
					^2^50/285				
RQ5	0.67	3.87	3.52	8	^1^170/170	601/632	11.0 ± 0.1	74	44
					^2^50/285				
RQ6	0.67	4.34	3.52	5	^1^170/170	592/626	10.3 ± 0.2	69	49
					^2^50/285				

### Zinc Oxide (ZnO) Synthesis

First, two separate solutions were prepared for synthesis: a 0.5 M TMAH solution in 10 mL of ethanol and a 0.1 M zinc acetate dihydrate solution in 30 mL of DMSO. Next, the TMAH solution was gradually added to the stirring zinc solution over the course of 2 min. After that, the stirring solution was allowed to react for 1 h under ambient conditions. Finally, it was precipitated with acetone and acetone/hexane, respectively, and dissolved in ethanol after each.

### QLED Fabrication

Glass substrates precoated with ITO were cleaned in an ultrasonic cleaner by using a mixture of soap and hot deionized (DI) water, followed by further washing via hot DI water and isopropyl alcohol, respectively. Then, the substrates were dried with nitrogen and plasma cleaned. On top of the ITO substrates, filtered PEDOT:PSS was spin-coated at 3500 rpm for 3 s and at 5000 rpm for 57 s to act as an efficient hole injection layer. After that, the substrates were annealed at 150 °C for 30 min. Then, they were transferred into the glovebox. The hole transport layer was spin-coated onto the PEDOT:PSS-coated substrates using a 6 mg mL^–1^ chloroform solution of PVK at 3000 rpm for 60 s; the substrates were then annealed at 150 °C for 30 min. InP/ZnSe/ZnS QDs (10 mg mL^–1^ in hexane) were dynamically spin-coated onto the PVK layer at 1250 rpm for 30 s to act as an emissive layer. For the electron transport layer, a 20 mg mL^–1^ ZnO solution was spin-coated at 2000 rpm for 60 s and annealed at 90 °C for 30 min. To complete the device fabrication, Al (100 nm) was thermally evaporated at a base pressure of 5 × 10^–6^ Torr to act as the cathode layer. Before the devices were taken for characterization, they were encapsulated with ultraviolet (UV)-curable epoxy and encapsulation glass.

### Characterizations

A set of characterization techniques was used to evaluate the structural and optical properties of the purified InP/ZnSeS/ZnS QDs and to measure the optoelectronic characteristics of the fabricated QLEDs. In this regard, X-ray diffraction (XRD) patterns (Bruker, D8 DISCOVER), scanning transmission electron microscopy (STEM) images and energy dispersive X-ray spectra (EDX, Thermo Fisher Scientific, measured via an ELECT plus detector and a Gemini 300 microscope), X-ray photoelectron spectra (XPS, Thermo Fisher Scientific Kα X-ray spectrometer), Fourier transform infrared spectra (FTIR, Nicolet 6700), UV–vis spectra (Thermo Fisher Scientific, GENESYS 10S), PL spectra (Cary Eclipse), time-resolved PL (TRPL) spectra (PicoQuant, FluoTime 200 time-correlated single photon counting (TCSPC) system), and PLQE measurements (Quantaurus-QY, Hamamatsu) were taken to analyze the purified QDs. The luminance, EQE, current density, and electroluminescence (EL) characteristics of the devices were measured using a Hamamatsu PMA-12 photonic multichannel analyzer and a Keithley 2400 source meter, together with an integrating sphere.

## Results and Discussion

**Figure 1:**
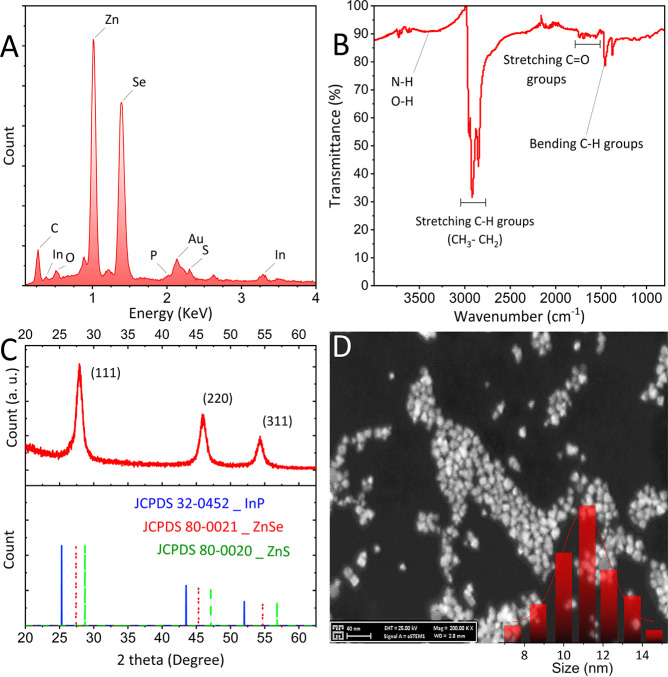
The (A) EDX profile, (B) FTIR spectrum, (C) XRD pattern, and (D) STEM image with a scale bar of 40 nm (inset shows the corresponding size histogram) of the purified InP/ZnSe/ZnS QDs (RQ5).

The EDX profile of the purified InP/ZnSe/ZnS QDs shows the presence of the In, P, and S elements and large amounts of the Zn and Se elements (Figure 1A). This confirms the presence of a thick ZnSe shell. The FTIR spectrum of sample RQ5 (Figure 1B) reveals a multipeak profile. An obvious peak at 1456 cm^–1^ is assigned to the scissoring vibrations of CH_2_.^26^ There are also very weak absorption bands of carboxylate stretching vibrations (C=O) at around 1560–1740 cm^–1^. This implies the partial functionality of C=O stretching groups on the surface of the QDs due to metal–sulfide bonds. Intense multiple peaks at around 2900 cm^–1^ are assigned to the symmetric and asymmetric vibrations of C–H via CH_2_ and CH_3_ groups.^27^ The wide signal at wavenumbers in the range of 3000–3500 cm^–1^ is attributed to the well-known hydrogen bridges of the O–H and N–H groups.

In Figure 1C, the XRD pattern indicates that the samples have been crystallized in a zinc blend cubic structure with the lattice plane Miller indices (111), (220), and (311). Considering the XRD patterns of the InP (JCPDS 32-0452), ZnSe (JCPDS 80-0021), and ZnS (JCPDS 80-0020) bulk structures that are indicated at the bottom of Figure 1C, the reflection peaks of the prepared QDs are between peaks related to ZnSe and ZnS.^28^ The pattern is closer to that of ZnSe due to the thick ZnSe layer coated around the InP QDs. To evaluate the morphology of the purified QDs, a STEM image was captured, which indicates the formation of tiny, three-dimensionally confined NCs with an average size of about 11.05 nm. The size histogram in the inset of Figure 1D also shows a relatively small size distribution.

**Figure 2:**
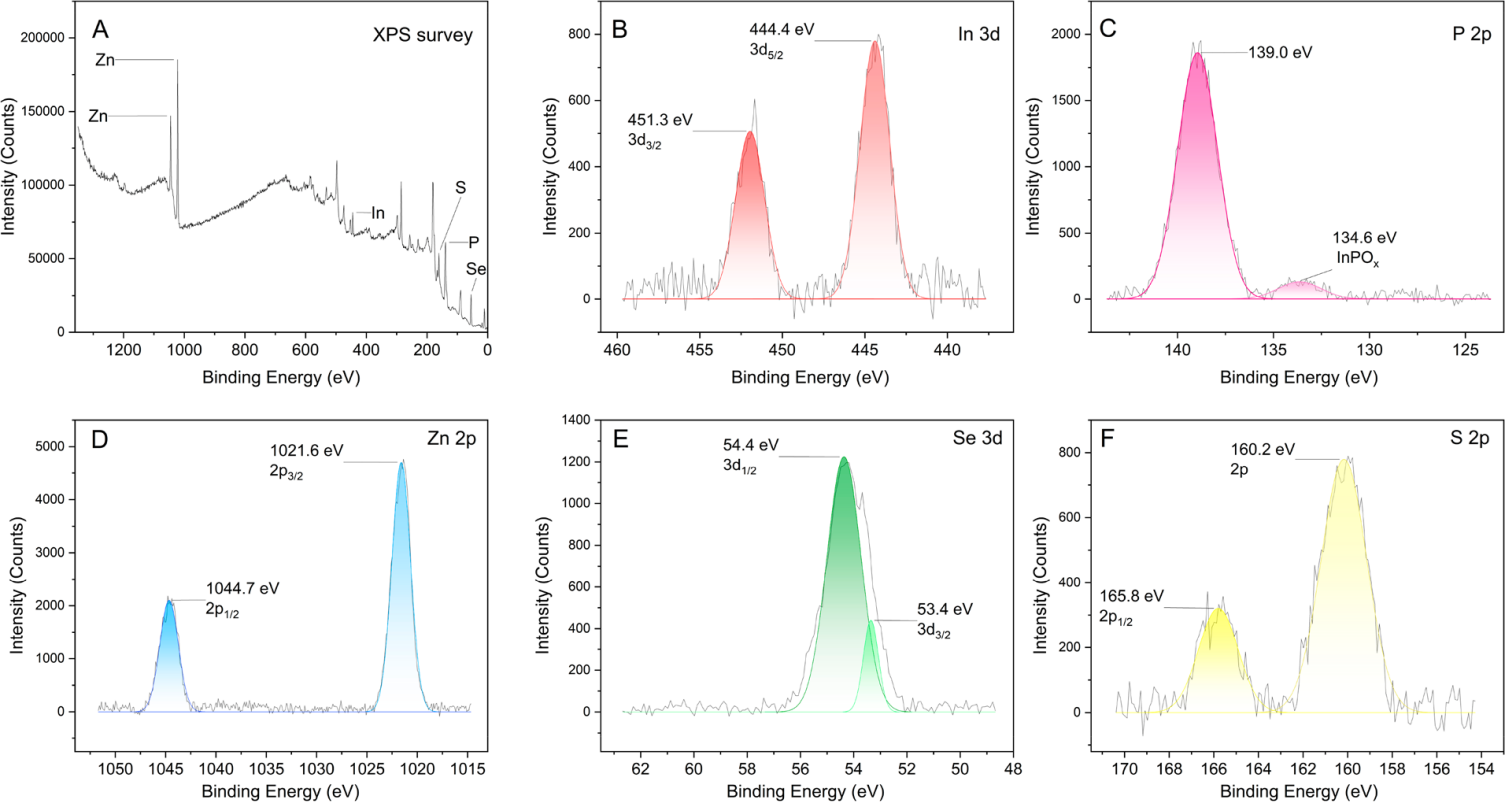
(A) XPS survey and (B–F) baseline-corrected high-resolution XPS results for each element in RQ5.

The XPS measurements for RQ5 show all of the expected elements (Figure 2). The high-resolution spectra in Figure 2B,C indicate two In-related peaks at 444.4 eV (3d_5/2_) and 451.3 eV (3d_3/2_), as well as an intense peak at 139.0 eV, which is assigned to P 2p.^21^ Importantly, the positive effect of the HF treatment is proven in Figure 2C, where a very weak InPO_*x*_ signal appears, demonstrating the effective etching role of the HF treatment. The intense peaks of Zn_2p_ and Se_3d_ seen in Figure 2D,E support the formation of the ZnSe shell layer with no evidence of the formation of Se–O species at 59 eV.^29^ The termination of the prepared QDs with a ZnS outer shell is also confirmed in Figure 2F via the S_2p_ signal without a signal related to surface oxidization. It should be noted that the obvious peak at 165.8 eV is related to Se_2p_, showing the formation of a thick ZnSe shell.

**Figure 3:**
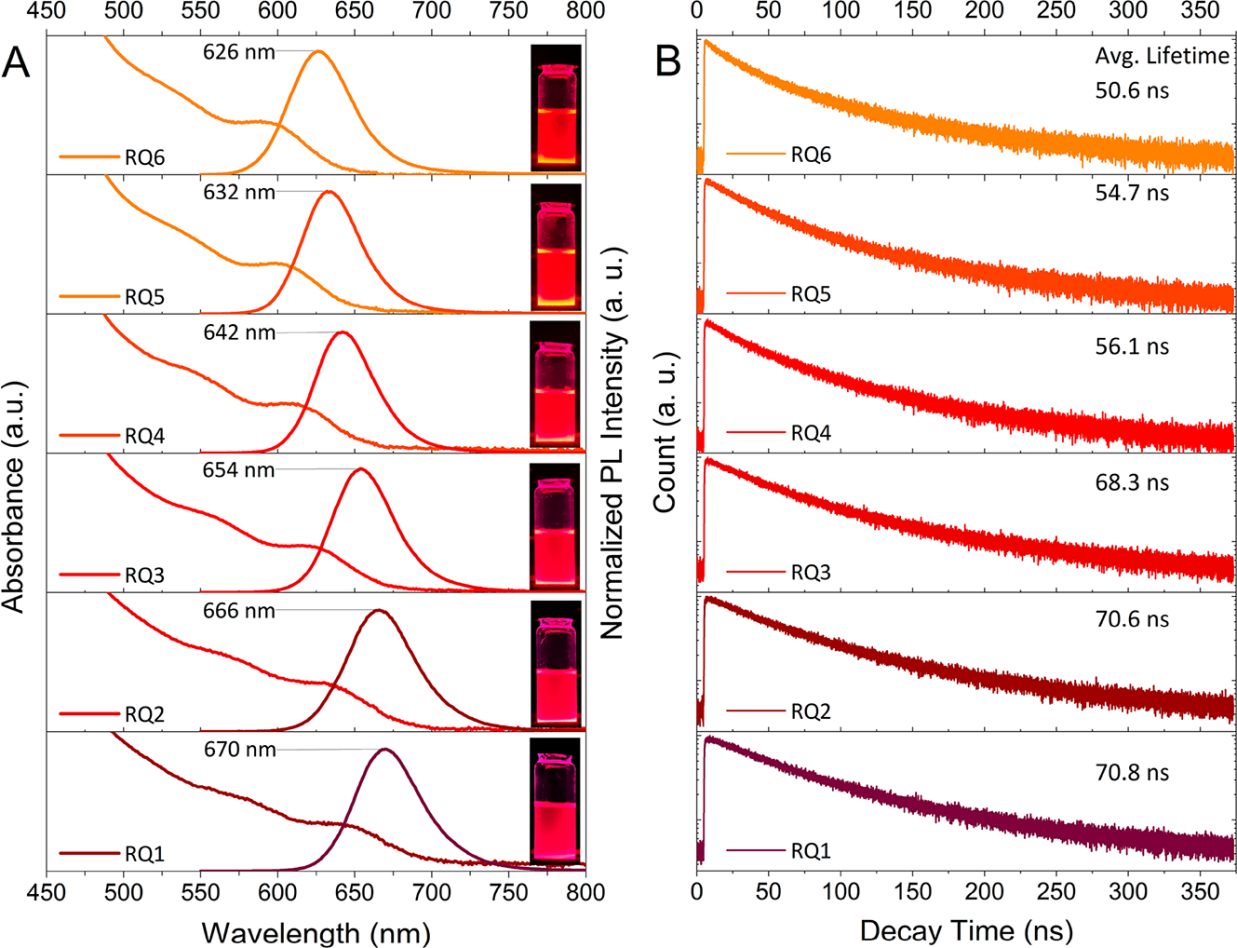
(A) UV–vis and PL spectra of the purified InP/ZnSe/ZnS QDs (insets: digital images of purified QDs dispersed in hexane under a 365 nm UV lamp). (B) TRPL profiles of the corresponding QDs.

The optical characteristics of the purified InP/ZnSe/ZnS QDs were evaluated by using a set of measurements to demonstrate the optical advantages of these QDs. Even though the synthesis process was a one-pot process, where a separate purification of the core QDs was not performed, the UV–vis spectra of all samples show two absorption peaks, thus proving the well-resolved and appropriate electronic structure of the prepared QDs (Figure 3A). These results also confirm a blue-shift in the first absorption peak from 645 nm (RQ1) to 595 nm (RQ6) upon changing the experimental parameters (Table 1 and Figure 3A). Simultanously, the PL emission spectra experiences a blue-shift from 670 nm (RQ1) to 626 nm (RQ6), fully covering the red region of the visible spectrum. In addition to the emission wavelength, the full width of the PL profiles of the prepared QDs is also in an excellent range, being as narrow as 44 nm for the RQ5 sample with its PL_peak_ located around 632 nm. In all cases, the Stokes shift is around 30 nm (between 28 and 34 nm for all samples), which shows the main role of excitonic states in the recombination of excited carriers and that it works for all samples. Interestingly, while the PL peak position was located in a wide range, the fwhm was reasonably narrow (50 nm) even for the RQ1 sample, with the PL emission located at the far-red region of 670 nm. As shown in Table 1, the recorded fwhm values of the PL emission spectra for all the samples were 44–52 nm, which are excellent results for such a one-pot approach. In the method employed here, the large amount of ZnCl_2_ that was used during the core synthesis provided a better situation to localize Zn^2+^ ions in the In_vacancy_ sites. At the same time, it was able to facilitate the formation of an efficient ZnSe shell layer around the core QDs, which resulted in better surface passivation. The subsequent injection of the Zn and Se precursors resulted in a thick ZnSe shell, which not only decreased the possibility of exciton leakage toward the surface of the QDs but also shifted the bandgap to longer wavelengths, thus facilitating access to the deep-red region with improved size homogeneity. This is demonstrated in Figure S1, where a similar synthesis method to RQ1 was repeated without the multistep addition of the ZnSe precursors. As can be seen, removing the thick ZnSe shell layer resulted in an obvious blue-shift from 670 to 643 nm. Therefore, in the absence of the thick ZnSe shell layer, we lose the ability to manipulate the experimental parameters and tune the emission wavelength across the entire red wavelength region (thus, the thick ZnSe shell layer shifts the PL emission to longer wavelengths). Additionally, the PL profile of the InP/ZnSe_thick_/ZnS QDs is narrower (50 nm) than that of the InP/ZnSe_thin_/ZnS QDs (58 nm). This result again demonstrates the key role of coating the core with a thick ZnSe layer for realizing narrow emissions, which is the ultimate goal in the synthesis of InP-based QDs. Even the absorption onset in the UV–vis absorbance spectrum is well-resolved in the case of a thick intermediate layer. In the end, it should be mentioned that the Stokes shift in the case of the thick layer is 28 nm, while it is around 39 nm for a thin layer of ZnSe. This implies the inappropriate passivation of midgap energy levels and the ensuing formation of deep trap states, as confirmed by the decrease in PLQE from 60% (ZnSe_thick_) to 43% (ZnSe_thin_). Therefore, such a thick intermediate shell layer is also beneficial for achieving tunable emission, decreasing the emission full width, and improving the emission purity. Furthermore, the large amount of OLA molecules that was used here may provide a dual positive effect. Primarily, due to its surfactant role, OLA can control the nucleation step (thus reducing the fast formation of the InP nuclei) and hinder the initial wide size distribution. At the same time, the reducing property of OLA can work against the formation of InPO_*x*_ states, thus facilitating the uniform growth of the core InP-based QDs. While the former decreases the size of the QD nuclei, the latter increases in the size of the QDs, both of which are beneficial for reaching a narrow emission profile in the presented study. OLA also works as a solvent in the synthesis method used here. This multifunctionality of OLA was confirmed via STEM images, where a nonlinear variation in the average size of the QDs was recorded (Figure S2). The captured STEM images show that the tunability of the emission profile in the samples RQ1–RQ6 is most likely not due to size effects. Instead, it is mainly attributed to the change in the composition of the cores (the increase in bandgap energy) due to the increase in the amount of Zn precursor used during the formation of the InP-based cores. In the case of RQ6, the large amount of ZnCl_2_ that was used may have increased the lattice mismatch, which, along with a very short reaction time, weakened the growth of QDs and dropped the average size of the QDs from 11.0 to 10.3 nm. To further demonstrate the high purity of the red color of the emissions, digital images of the purified QDs redispersed in hexane were captured under 365 nm irradiation (see the insets of Figure 3A). Independent of the emission wavelength, all of the QDs showed a pure-red emission. As seen in Figure S3, the InP/ZnSe/ZnS QDs have improved intensities under 365 nm UV irradiation, showing another synthetic advantage of the present method. Figure S3 shows the purified powder form of sample RQ5 synthesized via a single synthesis batch having pure-red emission. The preparation and purification processes that were used to produce this powder were exactly the same as for RQ5, except for a 6× increase in the amount of all reactants. To further reduce the full width of the samples, RQ5 was used for a size selection process using ethanol (details can be found in the Supporting Information). As seen in Figure S4, this size selection process resulted in a PL emission signal at 631 nm with a fwhm of 43 nm.

The PLQE of all samples was measured by the absolute method at an excitation wavelength of 320 nm. As mentioned in Table 1 and confirmed for RQ1 and RQ2 in Figure S5, the highest PLQE is 74% for RQ5, and it is maintained up to 60% even at long emission wavelengths of >665 nm. These can be considered as strong evidence for the functionality of the prepared QDs for the fabrication of red-emissive required systems. Table 2 summarizes recent high-quality reports on the one-pot colloidal synthesis of red InP-based QDs, demonstrating the narrow emission profile of the presented samples and comparing them to other one-pot approaches.

**Table 2:** Optical emission results of the purified red-emissive InP/ZnSe/ZnS QDs and comparison of their results with recent reports

composition	PL position (nm)	fwhm (nm)	PLQE (%)	publication year	ref
InP/ZnSeS/ZnS one-pot	616	63	81.8	2020	(22)
InP/ZnSeS one-pot	601	52	80	2021	(8)
InP/ZnSe/ZnS InP/ZnSe one-pot	607–615	45.5–47.5	90–95	2022	(15)
	614.5	44	52		
InP/ZnSe/ZnS one-pot InP/ZnSe/ZnS two-pot	620	59	82	2022	(6)
	620	49	90		
InP/ZnS one-pot	628	55	73	2022	(23)
InP/ZnSeS/ZnS one-pot	614	53.5	95.6	2022	(24)
InP/ZnSe/ZnSeS/ZnS one-pot	613	54	97.7	2022	(17)
InP/ZnSe/ZnSeS/ZnS one-pot	680	66	95	2023	(25)
InP/ZnSe/ZnS one-pot	632	43	74	present study	
	670	50	60		

To evaluate the number of energy levels contributing to the recombination of excited charge carriers, TRPL measurements were obtained via a TCSPC system. As shown in Figure 3B and confirmed via exponential fitting of each profile (Table 3), there are two radiation pathways that include fast and slow decay components, indicated with shorter (τ_1_) and longer (τ_2_) lifetimes, respectively. The shorter component is related to the recombination centers commonly known as midgap energy levels, while the longer component is due to the excitonic states involving radiative emissions. Interestingly, the latter component shows a long lifetime of up to 125 ns. The calculated average lifetimes of samples RQ1–RQ6 are 70.8, 70.6, 68.3, 56.1, 54.7, and 50.6 ns, respectively. This pattern indicates that, upon the blue-shift in the PL peak position, the average lifetime decreases, which can be attributed to an increase in the bandgap energy and a more dominant contribution of the localized energy levels in the bandgap.

**Table 3:** Lifetime components of the prepared InP/ZnSe/ZnS QDs

	RQ1 (670 nm)	RQ2	RQ3	RQ4	RQ5	RQ6 (626 nm)
τ_1_ (ns)	45.0	46.6	40.0	31.3	32.2	25.1
*A*_1_ (counts)	634.0 ± 16.0	580.8 ± 15.4	539.2 ± 16.9	524.6 ± 7.8	596.4 ± 19.1	500.1 ± 21.6
τ_2_ (ns)	125.1	115.0	112.6	94.2	96.5	85.5
*A*_2_ (counts)	301.1 ± 6.9	314.3 ± 7.3	344.4 ± 7.4	342.3 ± 7.8	321.6 ± 7.8	365.7 ± 8.3
τ_avg_ (ns)	70.8	70.6	68.3	56.1	54.7	50.6
χ^2^	0.991	0.990	0.996	1.020	1.002	1.001

**Figure 4:**
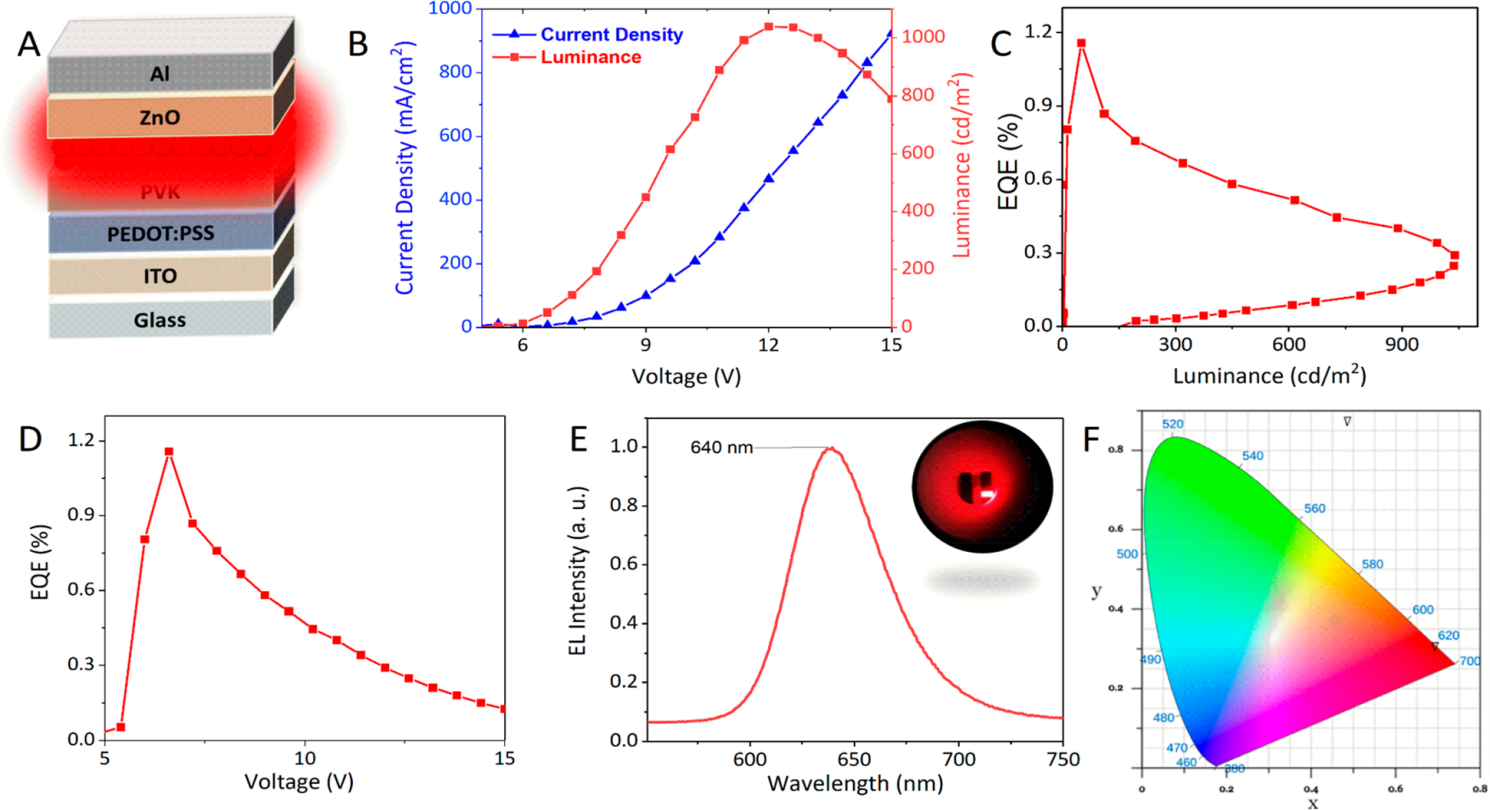
(A) QLED device configuration. (B) The *J*–*V*–*L* characteristic curves of the QLED. EQE versus (C) luminance and (D) voltage. (E) The EL spectrum of the QLED (inset: a photograph of the device at a driving voltage of 13 V). F) CIE coordinates of the QLED at a driving voltage of 12.6 V.

Next, using the InP/ZnSe/ZnS QDs (RQ5), we fabricated QLEDs comprising ITO/PEDOT:PSS/PVK/QDs/ZnO/Al. The device configuration is shown in Figure 4A. In Figure 4B, the current density–voltage–luminance (*J*–*V*–*L*) characteristics of the QLED shows that when the voltage is 12 V, the maximum brightness is 1039 cd m^–2^ and the current density is 465.5 mA cm^–2^. The turn-on voltage of the QLED was around 5 V. Figure 4C shows how the external quantum efficiency (EQE) changes with brightness. The EQE is at its maximum of 1.16% when the brightness is 50.64 cd m^–2^. When the brightness reaches its maximum value at 1039 cd m^–2^, the EQE is 0.29%. Figure 4D shows how the EQE changes as the driving voltage increases, and the EQE reaches a maximum of 1.16% at 6.6 V. The EL intensity peak at 640 nm at a driving voltage of 12.6 V is shown in Figure 4E, along with a photograph of the device at a driving voltage of 13 V (inset). Figure 4F shows that the CIE (*x*, *y*) coordinates (0.69, 0.30) are located in the deep-red region. It has been postulated that electron transport, which is influenced by the QD layers and shell thickness, is more important for determining the total current density.^13^ Furthermore, the EQE and luminescence of the LEDs were vastly affected by the thickness of the thick ZnSe interlayer. As has been previously found, a thick shell for Cd-based QDs helped prevent Auger recombination, which resulted in QD charging and a decrease in the device efficiency.^30^ The interparticle distance can be increased and the energy transmission between nearby QDs can be decreased by a thick shell.^31^

## Conclusion

The demand for the fabrication of efficient QLEDs with high-quality, stable, and less toxic QDs is at the heart of current research in optoelectronic devices. The present study suggested a facile one-pot colloidal method for preparing high-quality InP-based QDs with narrow emission within the red region of the visible spectrum. In summary, InP/ZnSe/ZnS QDs were synthesized by using HF-assisted surface fluorination and by implementing a thick midshell layer of ZnSe at high temperatures. By changing the experimental parameters, the fabricated QDs covered the entire red region of the visible spectrum from 626 to 670 nm, with the highest PLQE of 74% and a narrow fwhm of 43 nm. The QDs also demonstrated a PLQE of >66% for far-red emission wavelengths. To confirm the capability of these QDs in practical luminescent devices, we fabricated a QLED using the InP-based QDs as the emissive layer. This device had a maximum EQE of 1.16% and a maximum brightness of 1039 cd m^–2^ at a wavelength of 640 nm, which are values that are typically located at longer wavelengths when referencing other reports.

## Supporting Information Available

UV–vis and PL spectra of QDs prepared with a thin versus thick ZnSe shell layer, STEM images of the purified InP/ZnSe/ZnS QDs, photos of the large-scale synthesized InP/ZnSe/ZnS RQ5, PL spectra of RQ5 before and after the size selection process, and logarithmic plots of the absolute PLQE measurements for RQ1 and RQ2 (PDF)
