# Supplementary material for: Tuning the Shades of Red Emission in InP/ZnSe/ZnS Nanocrystals with Narrow Full Width for Fabrication of Light-Emitting Diodes
Source: ACS Omega. 2023 Oct 13;8(42):39690–8. doi: 10.1021/acsomega.3c05580 (PMC10600898; doi:10.1021/acsomega.3c05580)
Supplement: Supplementary file 1 — ao3c05580_si_001.pdf [file ao3c05580_si_001.pdf]

Supplementary Information

Tuning the Shades of Red Emission in InP/ZnSe/ZnS Nanocrystals with Narrow Full-width for Fabrication of Light-Emitting Diodes

Ehsan Soheyli[1*], Ayşenur Biçer[1], Sultan Suleyman Ozel[1], Kevser Sahin Tiras[2], Evren Mutlugun[1*]

1 Department of Electrical-Electronics Engineering, Abdullah Gul University, Kayseri 38080, Türkiye

2 Department of Physics, Faculty of Sciences, Erciyes University, Kayseri 38030, Türkiye

Corresponding Authors' Emails:

evren.mutlugun@agu.edu.tr

ehsan.soheyli@agu.edu.tr

A similar synthesis recipe to RQ1 ($InP/ZnSe_{thin}/ZnS$ QDs) was repeated, with just one round of injection of Se-TOP precursor to realize $InP/ZnSe_{thin}/ZnS$ QDs. Indeed, after the synthesis of core QDs, HF-acetone and 1Se-TOP was injected at 170 °C, followed by heating to 310 °C. At this temperature, ZnOAc (1.1 mmol ZnOAc dissolved in 1.6 mmol OA and 2 mL ODE) and S-TOP precursors were quickly injected into the stirring solution. After 30 min, the reaction cooled down to 210 °C, and 0.6 mL of OT was dropwisely added and the reaction continued for 1 h. In the end, the purification process was considered similar to the others.

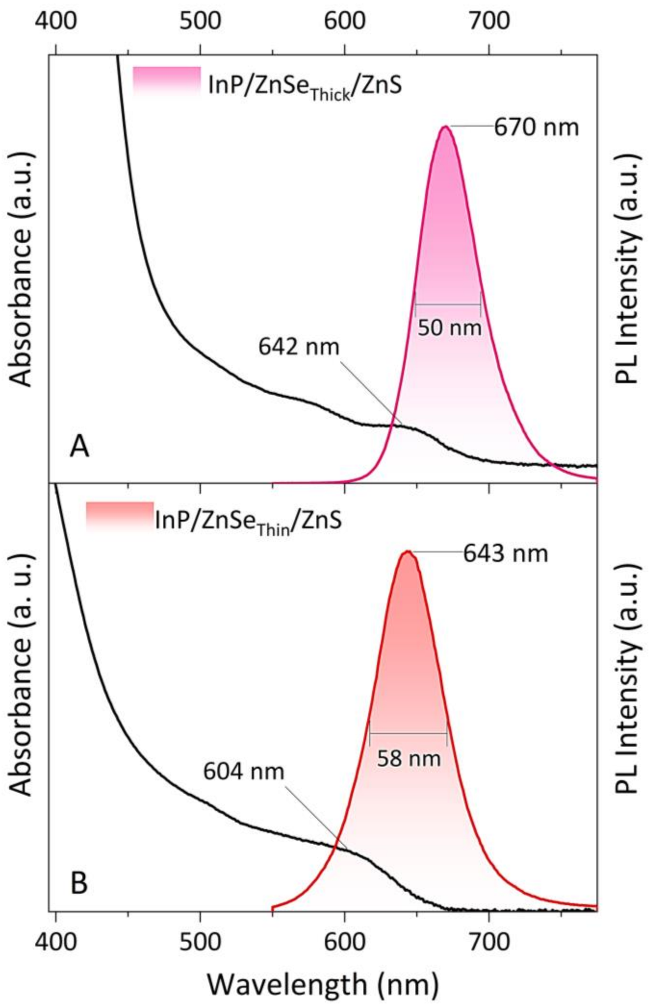

Figure S1: UV-Vis and PL spectra of (A) $InP/ZnSe_{thick}/ZnS$ and (B) $InP/ZnSe_{thin}/ZnS$ QDs.

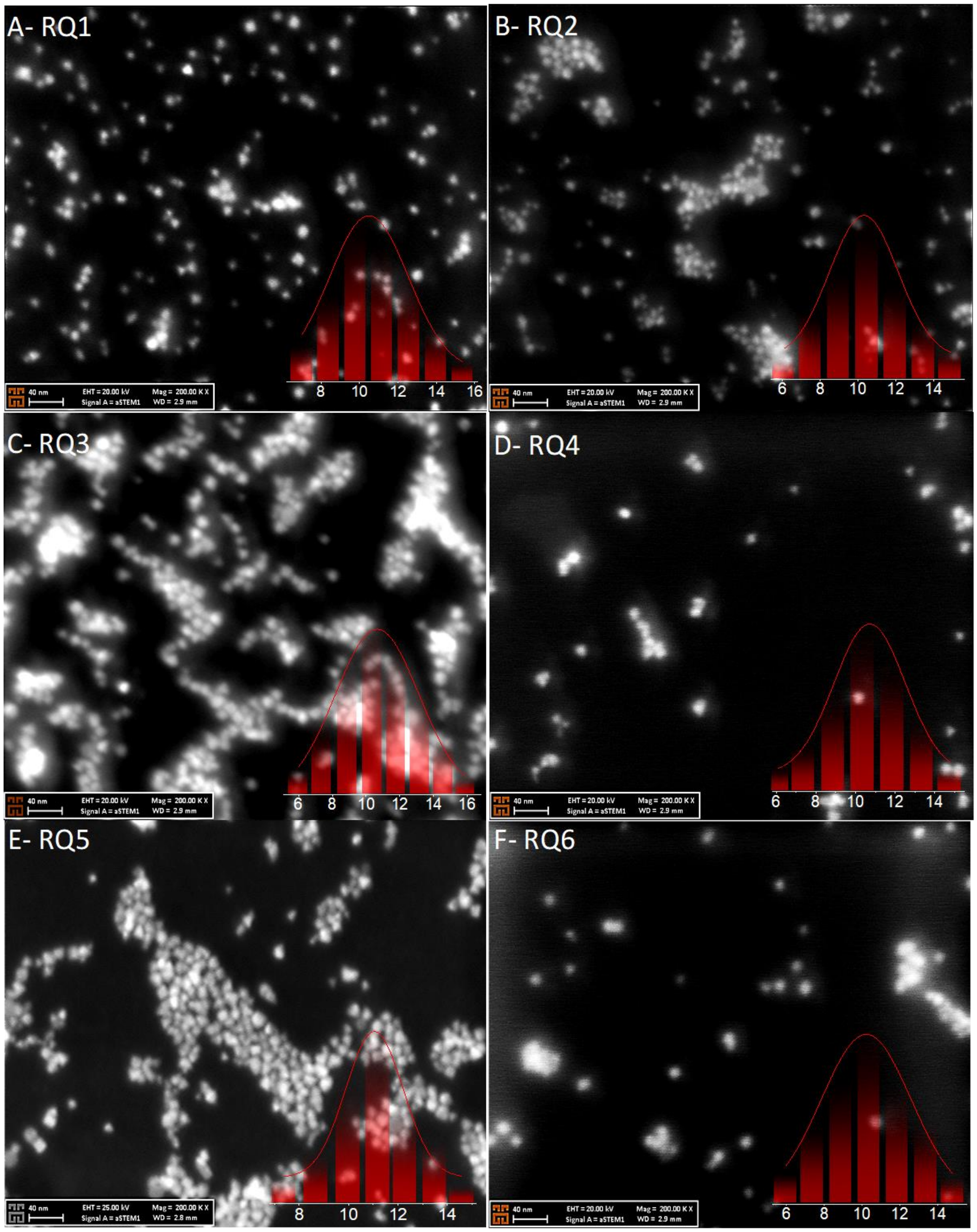

Figure S2: STEM images with a scale bar of 40 nm (inset shows corresponding size histogram) of purified InP/ZnSe/ZnS QDs.

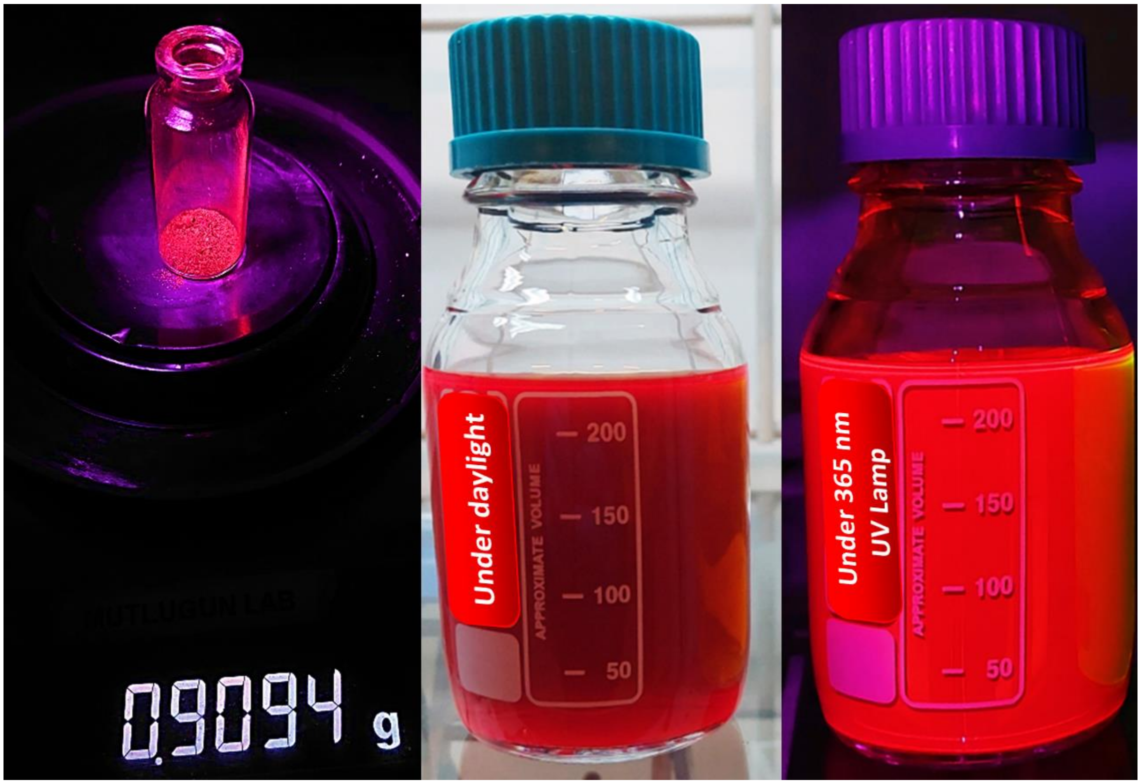

Figure S3: Photos of purified InP/ZnSe/ZnS RQ5 (powder and colloidal form) under a 365 nm UV lamp irradiation synthesized in large-scale.

The size-selection process was realized as follows: To remove the unreacted species, the same volume of hexane was added to the crude QDs solution and centrifuged at 4000 rpm for 5 min. The precipitated parts were removed. Again, hexane was added (3:1 vol ratio QDs:Hex). Next, ethanol as an antisolvent agent was dropwisely introduced into the mixture till oversized QDs were precipitated (typically around 1-1.5 mL). A successive centrifugation was realized at 4500 rpm for 10 min to separate the precipitates properly. Then, the supernatant was used for the second round of purification, adding less volume of ethanol. The same cycle of centrifugation (3500 rpm)/removing precipitate/adding ethanol (in less volume) was repeated three times more to improve the monodispersity of final QDs product.

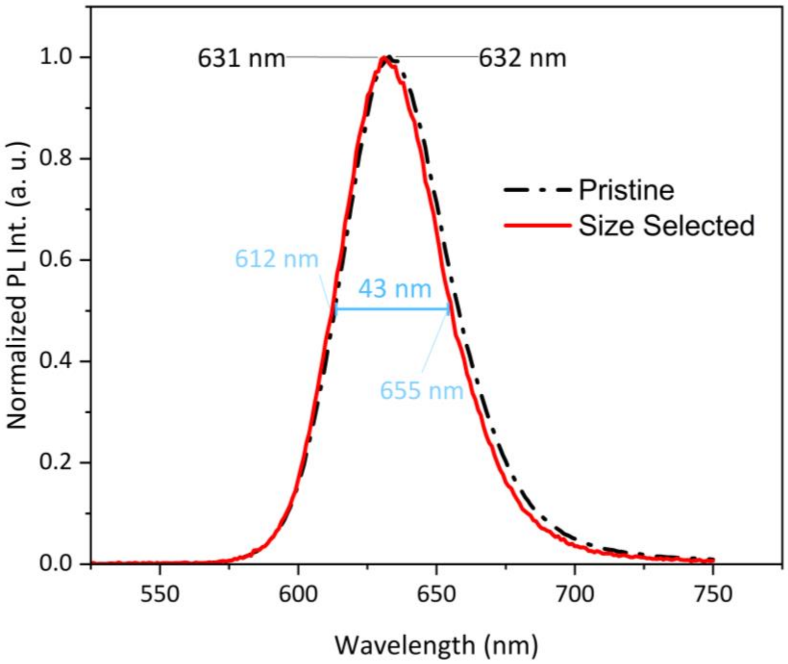

Figure S4: Normalized PL spectra of purified InP/ZnSe/ZnS RQ5 before and after the size selection process.

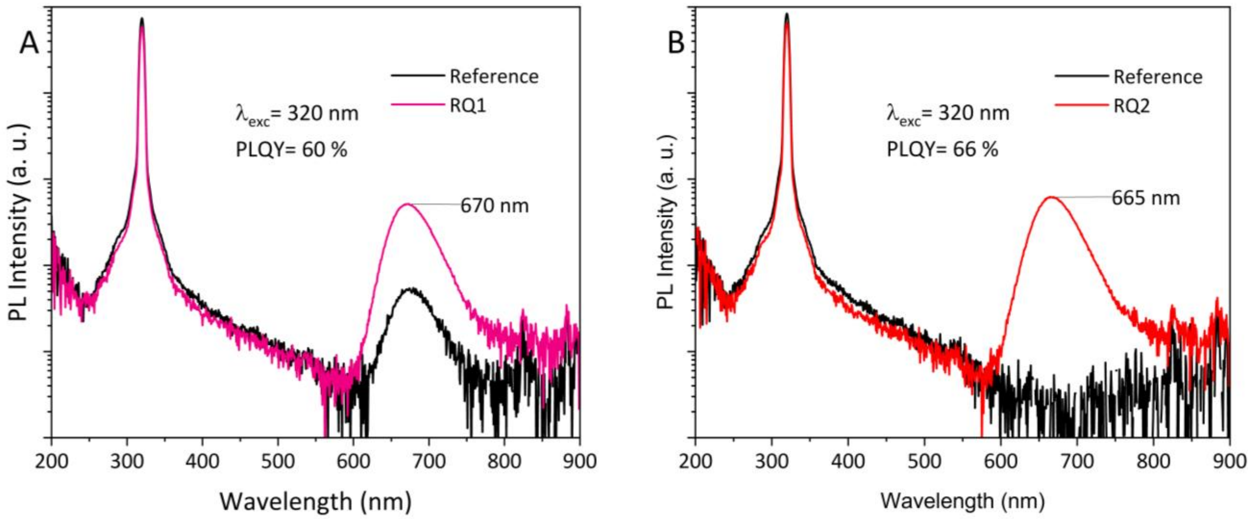

Figure S5: Logarithmic plots of absolute PLQE measurements for InP/ZnSe/ZnS; (A) RQ1 and (B) RQ2.
